# Diagnosis of *Centrocestus formosanus* Infection in Zebrafish (*Danio rerio*) in Italy: A Window to a New Globalization-Derived Invasive Microorganism

**DOI:** 10.3390/ani10030456

**Published:** 2020-03-09

**Authors:** Antonino Pace, Ludovico Dipineto, Serena Aceto, Maria Concetta Censullo, Maria Carmen Valoroso, Lorena Varriale, Laura Rinaldi, Lucia Francesca Menna, Alessandro Fioretti, Luca Borrelli

**Affiliations:** 1 Department of Veterinary Medicine and Animal Productions, Università degli Studi di Napoli Federico II, via Delpino 1, 80137 Naples, Italy; antonino.pace@unina.it (A.P.); ludovico.dipineto@unina.it (L.D.); lorena.varriale@unina.it (L.V.); laura.rinaldi@unina.it (L.R.); menna@unina.it (L.F.M.); fioretti@unina.it (A.F.); 2 Department of Biology, Università degli Studi di Napoli Federico II, via Cintia 26, 80126 Naples, Italy; serena.aceto@unina.it (S.A.); mariaconcettacensullo@gmail.com (M.C.C.); mariacarmen.valoroso@unina.it (M.C.V.)

**Keywords:** digenetic trematodes, gill fluke, invasive species, molecular diagnosis, species-specific primer pair, zoonosis

## Abstract

**Simple Summary:**

*Centrocestus formosanus* is an invasive parasite which originated from outside the European countries. Infections by this parasite seem to be related to the movements of its hosts. However, in Europe, the presence of *C. formosanus* has been sporadically reported and its zoonotic potential is still underestimated. Therefore, the present study proposes a fast and inexpensive diagnostic method through molecular analyses targeting the ribosomal internal transcribed sequence 2 (ITS2) using a newly designed species-specific primer pair. Given the potential negative consequences of *C. formosanus* global expansion, those responsible should adopt a one health approach to control the spread of this organism.

**Abstract:**

*Centrocestus formosanus* is a digenetic trematode with a complex life cycle, involving invertebrate and vertebrate hosts, humans included. In particular, it causes gill lesions and mortality in freshwater fish species, and gastrointestinal symptoms in infected humans. Here, we describe the occurrence of *C. formosanus* infection in zebrafish imported in Italy and propose a newly designed species-specific primer pair to ameliorate the diagnostic investigations for *C. formosanus*. Gill arches of 30 zebrafish were examined for the presence of encysted metacercariae under a stereomicroscope and processed through molecular analyses targeting the ribosomal internal transcribed sequence 2 (ITS2). Although *C. formosanus* distribution was originally restricted to Asia, it has been subsequently reported in new countries, revealing itself as an invasive species and raising important concerns for biodiversity, economy, scientific research, as well as animal and public health. Given the crucial role played by the ornamental fish industry in spreading this parasite, there is an urgent need for control measures to prevent the introduction and establishment of *C. formosanus* in non-endemic areas, including Europe. We also suggest developing new strategies in microbiology and epidemiology to better explore this new globalization-derived invasive species.

## 1. Introduction

Digenea include about 18,000 species of internal metazoan parasites that affect a wide variety of vertebrate and invertebrate hosts [1,2]. Digenetic trematode infections in animals and humans have attracted much attention from different disciplines, in particular veterinary medicine. Specifically, fish-borne zoonotic trematodes represent a concerning health issue in many Asian countries [2,3,4,5,6]. Indeed, 20 years ago, the World Health Organization estimated that more than 18 million people infected with fish-borne trematodes, and more than half a billion people at risk of infection worldwide [7,8].

Within fish-borne trematodes, *Centrocestus formosanus* is a small heterophyid fluke, described for the first time in Taiwan [9] and widely distributed in Asia [4,5,10,11,12,13,14,15]. Since the 1950s, several authors have reported this species in new continents, although its occurrence could still be underestimated [16,17,18,19,20,21,22]. *C. formosanus*, similar to other digenetic trematodes, exhibits a complex life cycle, as described by Nishigori [9]. The adults reside in the small intestine of vertebrate definitive hosts, such as birds and mammals. Eggs produced by adult trematodes hatch into miracidia which use a thiarid snail as their first intermediate host to develop into cercariae. Subsequently, free-swimming cercariae encyst in second intermediate fish hosts, specifically in the gills, where they develop into metacercariae. Piscivorous birds and mammals, ingesting the infected fish, complete the cycle [4,15,16,18,19,23]. Analogously, human infections can occur through consumption of raw or improperly cooked fish, containing metacercariae [12,15,24]. *C. formosanus* is a generalist parasite with a broad range of fish hosts, in which it causes severe gill lesions, respiratory disorders, loss of production, and death, giving rise to health and economic concerns in relation to wild, farmed (food and ornamental), and laboratory fish [9,17,18,19,25,26,27,28,29,30,31].

Among the numerous freshwater fish species affected by *C. formosanus* [23,25], zebrafish (*Danio rerio*) have been considered susceptible to infection, but only three reports have been described to date [10,21,32].

*Danio rerio* is a freshwater fish native to Asia, although it is widely distributed worldwide, probably due both to aquarists’ predilection and researchers’ interest as an animal model [32,33,34,35,36]. On the one hand, *D. rerio* is one of the top 30 species in the ornamental fish industry, whose on-going growth (approximately 120 countries and 1.5 billion ornamental fish per year) has led to difficulties in supply, traceability, sustainability, susceptibility to disease, and antibiotic resistance [35,37]. On the other hand, zebrafish have been increasingly used for scientific research, where an optimal health status is imperative to avoid invalidating the research [29,34,38,39].

Given the importance of *C. formosanus* infection and dissemination for animal and public health, and the implications for aquaculture, research, and food safety, epidemiological investigations should be conducted in new geographical regions, in order to implement or improve appropriate preventive and control measures in these areas, as well as health-monitoring programs in research facilities [18,29,39].

The present study reports on the occurrence of *C. formosanus* metacercariae in the gills of zebrafish previously intended for research. To the authors’ knowledge, this is the second report of *C. formosanus* infections in zebrafish imported in Italy [32]. Since the scarce and fragmentary data in the European literature [19,32,40] are probably due to underestimated and underdiagnosed infections, we propose to increase the awareness and ameliorate the diagnostic investigations, to shed light on this zoonosis, using a morphological and molecular approach. In particular, we propose for the first time a fast and specific diagnostic method based on a species-specific primer pair to detect the presence of this new invasive species in Italy.

## 2. Materials and Methods

### 2.1. Animal Maintenance

In 2013, before the application of the Italian Legislative Decree 26/2014 (regarding the implementation of Directive 2010/63/EU), 30 zebrafish were obtained from the ornamental fish trade and recruited for research activities. All fish were male and female adults (4 to 6 month old, at the time) of heterozygous “wild type” strain. During that period, fish were kept in recirculating systems with deionized water, housed in groups of ten per 30 L tank, following an acclimation period of two weeks [41]. Fish were fed twice daily with sterilized commercial food (Sera Vipagran, Germany). The room and water temperatures were maintained at 25 to 27 °C and illumination (1010 ± 88 lux) was provided by ceiling-mounted fluorescent light tubes (Aqueon, Franklin, WI, USA) on a 14-h cycle (D/N = 14:10 h), consistent with the standards of zebrafish care [38,42,43,44]. Fish were treated in accordance with the Directive of the European Parliament and of the Council on the Protection of Animals Used for Scientific Purposes (Directive 2010/63/EU) and in agreement with the Bioethical Committee of the University Federico II of Naples (authorization protocol number 47339-2013).

**Figure 1:**
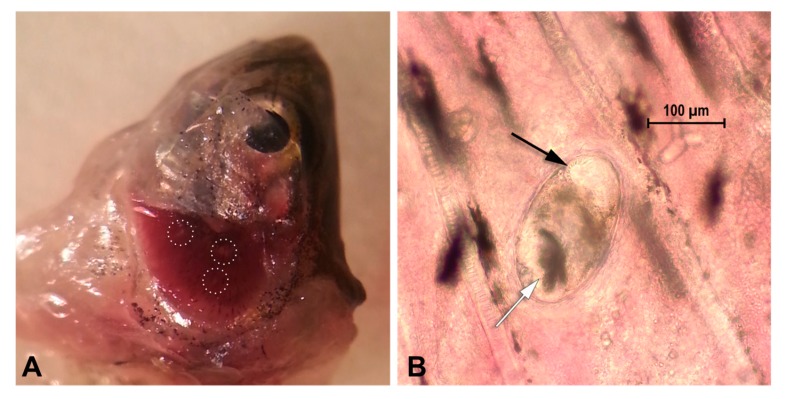
(**A**) Gills of an infected zebrafish. Parasitic cysts are visible as miliar white lesions on the gill tissue (dotted white lines); (**B**) Encysted metacercaria in the gill tissue of infected zebrafish. 40× microscopy evaluation. Note the X-shaped excretory bladder (white arrow) and part of the oral sucker (black arrow).

During standard physical examination, performed under anesthesia by immersion in 3-aminobenzoic acid ethyl ester (MS-222, Sigma–Aldrich, USA) at a concentration of 170 mg/L, buffered with sodium bicarbonate (1:2 ratio solution) [45,46], the gills of 20 zebrafish were found to be affected by small white spots, ascribable to parasitic cysts (Figure 1A). The animals were euthanized by immersion in an overdose of buffered MS-222 (500 mg/L) for at least 10 min following cessation of opercular movement, [33,45,46], but they had to be excluded from research activities.

### 2.2. Centrocestus Formosanus Examination and Identification

Fish bodies were dissected [38], gill arches were removed with the aid of a stereomicroscope (Leica, Milano, Italy) and prepared as wet mounts to be examined for the presence of encysted metacercariae [5,10,16,21,25,26,28,30,47]. Encysted metacercariae were examined under a light microscope (Leica, Milano, Italy) (Figure 1B) to evaluate their morphology and to be identified according to published characteristics [5,10,11,12,13,14,15,16,17,26]. Live encysted metacercariae were also recorded at 40× using a light microscope (Leica, Milano, Italy) (Supplementary Materials Video S1).

### 2.3. PCR Amplification and Sequencing

**Figure 2:**
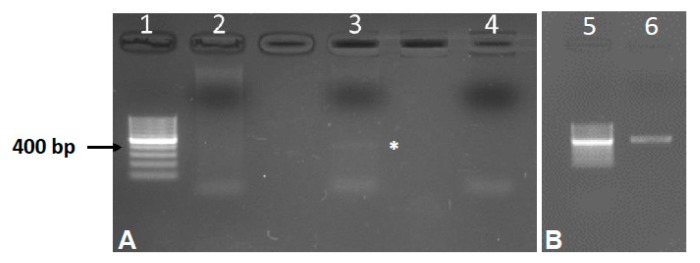
Agarose gel electrophoresis of the PCR amplification of DNA extracted from gills of *Danio rerio*. (**A**) PCR conducted using the primer pair ITS2_Centr_F/ITS2_Centr_R. (1) 100 bp ladder, (2) DNA extracted from uninfected gills, (3) DNA extracted from infected gills, (4) negative control (without DNA). The asterisk indicates the amplification fragment; (**B**) PCR conducted using the primer pair 3S/BD2. (5) 100 bp ladder and (6) DNA extracted from infected gills.

Total genomic DNA was extracted from 30 mg of gill tissue by using the QIAamp DNA Mini Kit (Qiagen, Hilden, Germany). DNA concentrations and quality were assessed by spectrophotometric measurements with a NanoDrop (ThermoFisher Scientific Inc., Waltham, MA, USA). Extracted DNA samples were stored at −80 °C until the molecular analyses described here. The detection of *C. formosanus* DNA was performed by PCR targeting the ribosomal internal transcribed sequence 2 (ITS2), using the primer pair 3S (5′-GGTACCGGTGGATCACTCGGCTCGTG-3′) and BD2 (5′-TATGCTTAAATTCAGCGGGT-3′), previously described [10,32]. As these primers are not specific for *C. formosanus*, we designed the species-specific reverse primer ITS2_Centr_R (5′-CGTGCAATGTTTGCATCGGA-3′) and used it together with the forward primer ITS2_Centr_F (5′-ATGAAGAGCGCAGCCAACT-3′) to amplify a 393 bp fragment of the ITS2 region of *C. formosanus*. PCR amplifications were conducted in the conditions previously described [10,32] and amplification products were visualized by 1.5% agarose gel electrophoresis (Figure 2). Subsequently, the amplicons were cloned into the pSC-A-amp/kan vector (Agilent), sequenced using the T3 and T7 plasmid primers and analyzed using an ABI 310 Genetic Analyzer (Applied Biosystems). The obtained sequences were examined through BLAST analysis.

## 3. Results

In this study, a total of 30 healthy zebrafish were examined and 20 showed miliar cystic gill lesions (Figure 1A). Cysts were small and elliptical. Inside the cysts, the coiled mature metacercariae were characterized by a large, dark, X-shaped excretory bladder (occupying the majority of the body caudal portion) and by approximately 32 circumoral spines surrounding the oral sucker, arranged in two rows (Video S1 and Figure 1B). Given the difficulty in counting the accurate number of circumoral spines, which is considered one of the most reliable characteristics in species identification, along with the excretory bladder, molecular analysis was conducted to confirm the taxonomic attribution. PCR resulted in the amplification of the ribosomal ITS2 region fragment. However, the nucleotide sequence of the amplicons obtained with the primer pair 3S/BD2 [10,32] corresponds to the ITS2 fragment of the host *Danio rerio*. On the contrary, the species-specific primer pair designed in the present work amplify a fragment of 393 bp showing ~99% nucleotide identity with the homolog ITS2 sequence of *C. formosanus* present in GenBank (Supplementary Materials Sequences S2).

## 4. Discussion

The occurrence of *C. formosanus* infection in zebrafish in Italy underlines the necessity to focus the attention on this invasive parasite, since this is the second case reported in the Italian peninsula [32]. Although its origin and distribution were initially restricted to Asia [4,9,10,11,12,13,24,31], *C. formosanus* has been subsequently reported in other countries, including Europe [16,17,18,19,20,21,22,25,26,29,48]. Actually, to the authors’ knowledge, this is the fourth case of *C. formosanus* infection in freshwater fish imported in Europe [19,32,40], confirming the introduction of this parasite in the European area, as well as the possible underestimation of this infection.

The causes of *C. formosanus* global spread are still subject to debate. Some authors pointed at the dissemination (deliberate or accidental) of its first intermediate host, *Melanoides tuberculata*, whereas others hold responsible the movements of birds and freshwater fish [16,17,19,21,25,27,28,30,49,50,51]. In particular, given the huge numbers of the ornamental fish industry in Europe (approximately 300 million fish, more than 2500 species, of which 60% are of freshwater origin), the importation of ornamental freshwater fish, from countries where *C. formosanus* is endemic, plays a crucial role in spreading this parasite [35,36,51]. Indeed, Asian countries are the major traders and most of the suppliers of freshwater fish to Europe are involved with *C. formosanus* infections [4,5,10,11,12,35,36,51].

The importance of *C. formosanus* dissemination is related to its ability to infect valuable fish species (ecologically or commercially), as well as humans [12,15,24,25,26,30]. In fish, the gill lesions caused by *C. formosanus* metacercariae eventually lead to respiratory difficulties and death, negatively affecting animal welfare, reducing productions in fish farms, and threatening biodiversity [4,5,17,19,22,26,28,30]. In humans, cases of *C. formosanus* co-infections were reported in Asia, with symptoms varying from epigastric pain to indigestion, occasionally accompanied with diarrhea [9,12,27]. Although to date there are no human cases in Europe, further investigations should be conducted, in order to keep a high level of attention on this issue [5,12,17,19].

Another insidious aspect is the impact of diseases of laboratory animals on the outcomes of research activities. Indeed, pathogens could confound experimental results or force researchers to exclude infected specimens from experimental trials [29,39], such as *C. formosanus* in the case presented here. The potential damage to research, in terms of costs and time, strengthens the necessity to improve our knowledge and develop measures to document and control diseases of fish used for experimental procedures, as well as implement appropriate health-monitoring programs in research facilities [29,39]. Actually, zebrafish involved in research activities can no longer be obtained from the ornamental industry, but from fish facilities specifically authorized for production (EU Directive 63/2010) [29]. Moreover, several zebrafish stock centers are now able to provide healthy and genetically characterized fish (e.g., the European Zebrafish Research Center in Germany; the Zebrafish International Resource Center in the USA, the two Taiwan Zebrafish Core Facilities, and the China Zebrafish Resource Center). These facilities, along with the EU Directive 63/2010, transposed in Italy after the collection of data reported here, should contribute to preserving the health status of laboratory animals, as well as the quality of science [29].

Concerning the diagnosis of *C. formosanus*, the number of circumoral spines has been acknowledged as a reliable criterion for species identification; however, morphological characteristics could be similar in larval stages, the accurate count of circumoral spines could be challenging, and their number might vary in different fish species [10,11,17]. Therefore, parasite identification should no longer rely only on standard morphological methods, but molecular analyses should be accepted as one of the most effective and accurate approaches for species confirmation [10,11,32]. For these reasons, we propose a fast, cheap and specific PCR-based method to address *C. formosanus* infection in zebrafish, starting from small pieces of the hosts’ gill tissue and avoiding elaborate collection of metacercariae. The parasite-specific primer pair eludes the frequent problem of amplifying the host DNA and makes this method also suitable for the detection of this invasive parasite in other potential hosts. The primer pair 3s/BD2 is able to amplify the ITS2 of *C. formosanus* [10,32]; however, it can also amplify the ITS2 of *Danio rerio*. The PCR amplification using the primer pair 3s/BD2, applied on the DNA extracted from our infected gill samples (containing both DNA of *Danio rerio* and metacercariae), resulted in the preferential amplification of the zebrafish ITS2. On the contrary, the species-specific primer pair designed in the present work is able to specifically amplify a fragment of the ITS2 region of *C. formosanus* (Figure 2), which does not have nucleotide similarity with any other species.

The current report draws the attention on *C. formosanus* as an invasive parasite, as well as on other species that could be similarly introduced in Europe, underlining the need for epidemiological studies and appropriate preventive and control programs, in order to monitor their occurrence and prevent their negative consequences for economy, biodiversity, scientific research, animal and public health [6,17,19,20,22,23,28,29,30,47,48]. Some aspects of biology and epidemiology, regarding both animal and human hosts, should be further explored in Europe [7,17,20,26,47,49]. Teams of experts in “one health” control should be the first actors involved in applying good management and efficient measures, especially during the intentional movement of animals, such as border inspection, accompanying health certification, quarantine measures, and, if necessary, treatment (prior to export or upon arrival) and disinfection procedures [19,22,29,48]. Additionally, adequate strategies should be applied in aquaculture facilities, including training of traders and farmers, regular examination of farmed fish, elimination of snail populations, avoidance of dispersal of farmed fish, and prevention of access to other species, especially birds and mammals [10,17,18,19,21,22,49]. Specific recommendations concerning diseases in ornamental fish should be strictly followed and the mobilization of these animals should be better considered on the basis of good practices and proper diagnosis of these potential pathogens [52]. This could preserve animal and human international health, limiting the introduction and transfer of zoonotic agents.

## 5. Conclusions

This work intends to highlight the importance of *C. formosanus*, whose zoonotic potential should not be underestimated, especially in non-endemic areas. We designed and proposed a species-specific primer pair to ameliorate the diagnostic investigations for this invasive parasite. Given the potential negative consequences of *C. formosanus* global expansion, those involved in fish farming and trading should adopt a “one health” approach to control the spread of this organism. We also suggest developing new strategies in microbiology and epidemiology to better explore this new globalization-derived invasive species.

## Supplementary Materials

The following are available online at https://www.mdpi.com/2076-2615/10/3/456/s1, Video S1: Live metacercaria encysted in the gill tissue of infected zebrafish, recorded at 40× using a Leica light microscope, Sequences S2: ITS2 sequences of C. formosanus.
