# Supplementary material for: Diagnosis of Centrocestus formosanus Infection in Zebrafish (Danio rerio) in Italy: A Window to a New Globalization-Derived Invasive Microorganism
Source: Animals (Basel). 2020 Mar 9;10(3):456. doi: 10.3390/ani10030456 (PMC7143865; doi:10.3390/ani10030456)
Supplement: Supplementary file 1 [file animals-10-00456-s001.zip › animals-729441-supplementary/Sequences S2.pdf]

ITS2 sequences of C. formosanus

>Centrocestus_formosanus_infecting_zebrafish
ATGAAGAGCGCAGCCAACTGTGTGAATTAATGTGAACTGCATACTGCTTTGAACATCGAC
ATCTTGAACGCACATTGCGGCCATGGGTTTTCCTGTGGCCACGCCTGTCCGAGGGTCGGC
TTATAAACTATCACGACGCCCAAAAAGTCGTGGCTTGGGTCTTGCCAGCCGGCGTGATTT
CCTTGTGCTTTGCATGGGGTGCCGGATCTATGGCTTTTCCCTAATGTGCCGGACGCAACC
ATCTCCAGGCTGGCGGTCTGGATGAGGAAGTGGCGGCGGAGTCGTGGCTCAATGATACAT
ATATATATATATATAATGCGCGCTCCGTTGTCTATTCCTTGTCTATGATCTCGGCATTGG
GTTTGGCAATGCATCCGATGCAAACATTGCACG

">KX430147.1 Centrocestus formosanus voucher CEN-ES 2016 5.8S ribosomal RNA gene, partial sequence; internal transcribed spacer 2, complete sequence; and large subunit ribosomal RNA gene, partial sequence"
ATGAAGAGCGCAGCCAACTGTGTGAATTAATGTGAACTGCATACTGCTTTGAACATCGAC
ATCTTGAACGCACATTGCGGCCATGGGTTTTCCTGTGGCCACGCCTGTCCGAGGGTCGGC
TTATAAACTATCACGACGCCCAAAAAGTCGTGGCTTGGGTCTTGCCAGCCGGCGTGATTT
CCTTGTGCTTTGCATGGGGTGCCGGATCTATGGCTTTTCCCTAATGTGCCGGACGCAACC
ATCTCCAGGCTGGCGGTCTGGATGAGGAAGTGGCGGCGGAGTCGTGGCTCAATGATACAT
ATATATATATA--TAATGCGCGCTCCGTTGTCTATTCCTTGTCTGTGATCTCGGCATTGG
GTTTGGCAATGCATCCGATGCAAACATTGCACG

">KX430148.1 Centrocestus formosanus voucher CEN-PU 2016 5.8S ribosomal RNA gene, partial sequence; internal transcribed spacer 2, complete sequence; and large subunit ribosomal RNA gene, partial sequence"
ATGAAGAGCGCAGCCAACTGTGTGAATTAATGTGAACTGCATACTGCTTTGAACATCGAC
ATCTTGAACGCACATTGCGGCCATGGGTTTTCCTGTGGCCACGCCTGTCCGAGGGTCGGC
TTATAAACTATCACGACGCCCAAAAAGTCGTGGCTTGGGTCTTGCCAGCCGGCGTGATTT
CCTTGTGCTTTGCATGGGGTGCCGGATCTATGGCTTTTCCCTAATGTGCCGGACGCAACC
ATCTCCAGGCTGGCGGTCTGGATGAGGAAGTGGCGGCGGAGTCGTGGCTCAATGATACAT
ATATATATATA--TAATGCGCGCTCCGTTGTCTATTCCTTGTCTGTGATCTCGGCATTGG
GTTTGGCAATGCATCCGATGCAAACATTGCACG

">KX430149.1 Centrocestus formosanus voucher CEN-AN 2016 5.8S ribosomal RNA gene, partial sequence; internal transcribed spacer 2, complete sequence; and large subunit ribosomal RNA gene, partial sequence"
ATGAAGAGCGCAGCCAACTGTGTGAATTAATGTGAACTGCATACTGCTTTGAACATCGAC
ATCTTGAACGCACATTGCGGCCATGGGTTTTCCTGTGGCCACGCCTGTCCGAGGGTCGGC
TTATAAACTATCACGACGCCCAAAAAGTCGTGGCTTGGGTCTTGCCAGCCGGCGTGATTT
CCTTGTGCTTTGCATGGGGTGCCGGATCTATGGCTTTTCCCTAATGTGCCGGACGCAACC
ATCTCCAGGCTGGCGGTCTGGATGAGGAAGTGGCGGCGGAGTCGTGGCTCAATGATACAT
ATATATATATA--TAATGCGCGCTCCGTTGTCTATTCCTTGTCTGTGATCTCGGCATTGG
GTTTGGCAATGCATCCGATGCAAACATTGCACG

">KX430150.1 Centrocestus formosanus voucher CEN-PE 2016 5.8S ribosomal RNA gene, partial sequence; internal transcribed spacer 2, complete sequence; and large subunit ribosomal RNA gene, partial sequence"
ATGAAGAGCGCAGCCAACTGTGTGAATTAATGTGAACTGCATACTGCTTTGAACATCGAC
ATCTTGAACGCACATTGCGGCCATGGGTTTTCCTGTGGCCACGCCTGTCCGAGGGTCGGC
TTATAAACTATCACGACGCCCAAAAAGTCGTGGCTTGGGTCTTGCCAGCCGGCGTGATTT
CCTTGTGCTTTGCATGGGGTGCCGGATCTATGGCTTTTCCCTAATGTGCCGGACGCAACC
ATCTCCAGGCTGGCGGTCTGGATGAGGAAGTGGCGGCGGAGTCGTGGCTCAATGATACAT
ATATATATATA--TAATGCGCGCTCCGTTGTCTATTCCTTGTCTGTGATCTCGGCATTGG
GTTTGGCAATGCATCCGATGCAAACATTGCACG

">KY075663.1 Centrocestus formosanus isolate 27.1 internal transcribed spacer 1, 5.8S ribosomal RNA gene, and internal transcribed spacer 2, complete sequence; and 28S ribosomal RNA gene, partial sequence"

ATGAAGAGCGCAGCCAACTGTGTGAATTAATGTGAACTGCATACTGCTTTGAACATCGAC
ATCTTGAACGCACATTGCGGCCATGGGTTTTCCTGTGGCCACGCCTGTCCGAGGGTCGGC
TTATAAACTATCACGACGCCCAAAAAGTCGTGGCTTGGGTCTTGCCAGCCGGCGTGATTT
CCTTGTGCTTTGCATGGGGTGCCGGATCTATGGCTTTTCCCTAATGTGCCGGACGCAACC
ATCTCCAGGCTGGCGGTCTGGATGAGGAAGTGGCGGCGGAGTCGTGGCTCAATGATACAT
ATATATATATACATAATGCGCGCTCCGTTGTCTATTCCTCGTCTGTGATCTCGGCATTGG
GTTTGGCAATGCATCCGATGCAAACATTGCACG

">KY075664.1 Centrocestus formosanus isolate 27.3 internal transcribed spacer 1, 5.8S ribosomal RNA gene, and internal transcribed spacer 2, complete sequence; and 28S ribosomal RNA gene, partial sequence"
ATGAAGAGCGCAGCCAACTGTGTGAATTAATGTGAACTGCATACTGCTTTGAACATCGAC
ATCTTGAACGCACATTGCGGCCATGGGTTTTCCTGTGGCCACGCCTGTCCGAGGGTCGGC
TTATAAACTATCACGACGCCCAAAAAGTCGTGGCTTGGGTCTTGCCAGCCGGCGTGATTT
CCTTGTGCTTTGCATGGGGTGCCGGATCTATGGCTTTTCCCTAATGTGCCGGACGCAACC
ATCTCCAGGCTGGCGGTCTGGATGAGGAAGTGGCGGCGGAGTCGTGGCTCAATGATACAT
ATATATATATACATAATGCGCGCTCCGTTGTCTATTCCTCGTCTGTGATCTCGGCATTGG
GTTTGGCAATGCATCCGATGCAAACATTGCACG

">KY075665.1 Centrocestus formosanus isolate 33.3 internal transcribed spacer 1, 5.8S ribosomal RNA gene, and internal transcribed spacer 2, complete sequence; and 28S ribosomal RNA gene, partial sequence"
ATGAAGAGCGCAGCCAACTGTGTGAATTAATGTGAACTGCATACTGCTTTGAACATCGAC
ATCTTGAACGCACATTGCGGCCATGGGTTTTCCTGTGGCCACGCCTGTCCGAGGGTCGGC
TTATAAACTATCACGACGCCCAAAAAGTCGTGGCTTGGGTCTTGCCAGCCGGCGTGATTT
CCTTGTGCTTTGCATGGGGTGCCGGATCTATGGCTTTTCCCTAATGTGCCGGACGCAACC
ATCTCCAGGCTGGCGGTCTGGATGAGGAAGTGGCGGCGGAGTCGTGGCTCAATGATACAT
ATATATATATA--TAATGCGCGCTCCGTTGTCTATTCCTTGTCTGTGATCTCGGCATTGG
GTTTGGCAATGCATCCGATGCAAACATTGCACG
