# Supplementary figures and images for: Diagnosis of Centrocestus formosanus Infection in Zebrafish (Danio rerio) in Italy: A Window to a New Globalization-Derived Invasive Microorganism
Source: Animals (Basel). 2020 Mar 9;10(3):456. doi: 10.3390/ani10030456 (PMC7143865; doi:10.3390/ani10030456)

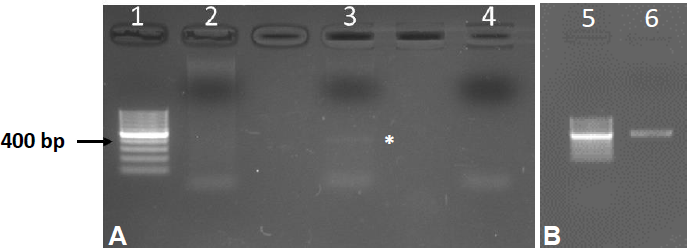

Supplement: Supplementary file 1 [file animals-10-00456-s001.zip › animals-729441-supplementary/Figure_2AB.tif]

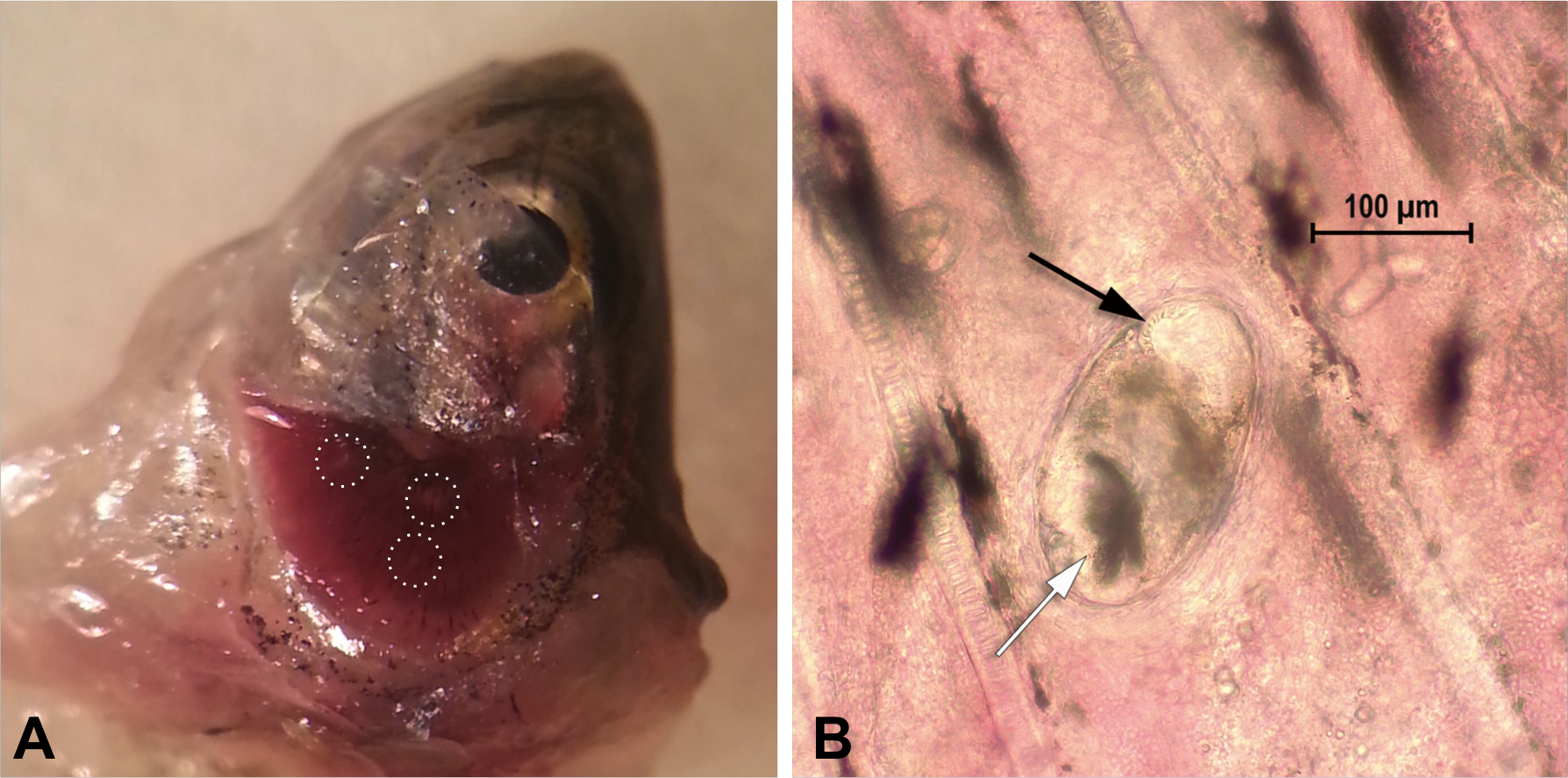

Supplement: Supplementary file 1 [file animals-10-00456-s001.zip › animals-729441-supplementary/Figure_1AB.tiff]
